# Frequency and Nuisance Level of Adverse Events in Individuals Receiving Homologous and Heterologous COVID-19 Booster Vaccine

**DOI:** 10.3390/vaccines10050754

**Published:** 2022-05-11

**Authors:** Piotr Rzymski, Dominika Sikora, Joanna Zeyland, Barbara Poniedziałek, Dorota Kiedik, Halina Falfushynska, Andrzej Fal

**Affiliations:** 1 Department of Environmental Medicine, Poznan University of Medical Sciences, 60-806 Poznań, Poland; dominika.sikora97@o2.pl (D.S.); bpon@ump.edu.pl (B.P.); 2 Integrated Science Association (ISA), Universal Scientific Education and Research Network (USERN), 60-806 Poznań, Poland; 3 Doctoral School, Poznan University of Medical Sciences, Fredry St. 10, 61-701 Poznań, Poland; 4 Department of Biochemistry and Biotechnology, Poznań University of Life Sciences, 60-632 Poznań, Poland; joanna.zeyland@up.poznan.pl; 5 Department of Population Health, Division of Public Health, Wroclaw Medical University, 50-345 Wroclaw, Poland; dorota.kiedik@umw.edu.pl; 6 Department of Orthopedagogy and Physical Therapy, Ternopil V. Hnatiuk National Pedagogical University, 46027 Ternopil, Ukraine; falfushynska@tnpu.edu.ua; 7 Collegium Medicum, Warsaw Faculty of Medicine, Cardinal Stefan Wyszyński University, 01-938 Warsaw, Poland

**Keywords:** SARS-CoV-2, pandemic, mRNA vaccines, vector vaccines, massive vaccinations, immunology

## Abstract

This study aimed to compare the occurrence and nuisance of adverse events following administration of each COVID-19 vaccine dose between two groups: individuals given three doses of mRNA vaccine (homologous group, 3 × mRNA, *n* = 252) and those given two doses of adenoviral vector vaccine further boosted with mRNA vaccine (heterologous group, 2 × AZ + 1 × mRNA, *n* = 205). Although the studied groups differed significantly in the frequency and number of side effects after the first and second vaccine dose, no relevant differences were seen following the booster administration. Arm pain and fatigue were the most common effects, regardless of the vaccination group and vaccine dose. In the homologous group, female sex, lower BMI, and no history of regular influenza vaccination were associated with a higher frequency of side effects of a booster dose. In the heterologous group, the history of COVID-19 was associated with an increased number of side effects seen after a booster. In both groups, the number of side effects related to the first and second dose correlated with the number observed after administration of a booster dose. Individuals receiving a homologous booster reported a higher nuisance of side effects than the heterologous group. It was similar to the level reported after the second dose in both groups. The use of pharmaceuticals to counteract the side effects was more frequent after a first dose in the 2 × AZ + 1 × mRNA group, but higher after second dose in individuals receiving the 3 × mRNA vaccination scheme. The frequency of pharmaceutical use after a booster dose was similar in both groups (approx. 60%). Paracetamol was most frequently chosen, regardless of the group and vaccine dose. In addition, the vast majority of participants (93%) declared to accept future doses of the COVID-19 vaccine if their administration would be recommended. This study provides an overview of the response to homologous and heterologous mRNA vaccine booster dose that may be valuable in shaping accurate and honest communication with vaccinated individuals, especially in those regions which are yet to pursue booster strategies.

## 1. Introduction

Vaccination is an important tool for decreasing the rates of COVID-19 hospitalization, ease the general overwhelming of the healthcare system, and suppress the SARS-CoV-2 evolution [1,2,3]. In response to the emergence of the novel, more transmissible viral variants, such as B.1.617.2 (delta) and later, B.1.1.529 (omicron), booster strategies have been adopted in various world regions [4,5,6,7]. With various COVID-19 vaccines authorized for use in different world regions [8,9], this has resulted in various combinations of primary and booster vaccinations [10]. It was partially driven by the varying availability of particular vaccines and contracts undersigned in specific regions with selected pharmaceutical companies. In the European Union, the booster programs were primarily based on the mRNA vaccines, i.e., BNT162b2 (BioNTech/Pfizer, Mainz, Germany/New York, NY, USA) and mRNA-1273 (Moderna Therapeutics, Cambridge, MA, USA), administrated after the second dose of the mRNA vaccine or the adenoviral vector vaccine AZD1222 (Oxford/AstraZeneca, UK/Sweden), or a single dose of Ad26.COV2.S (Janssen/Johnson & Johnson, Beerse, Belgium/New Brunswick, NJ, USA) [11]. In addition, the European Medicines Agency has also recommended Ad26.COV2.S as a booster vaccine [12].

An increasing number of clinical trials and observational studies have suggested that a heterologous vaccine booster dose (e.g., using the mRNA vaccine after a primary vaccination scheme based on the adenoviral vector vaccine) is generally well-tolerated. Moreover, they evoke robust immunity and, in some cases, may offer additional benefits in terms of greater humoral and T-cell response and, subsequently, higher effectiveness [13,14,15,16,17].

The data on the reactogenicity of heterologous booster vaccinations compared to homologous schedules are limited. However, some previous studies have shown that receiving the mRNA vaccine after the first dose of adenoviral vector vaccine, or vice versa, may lead to a higher frequency of side effects, mostly mild or moderate, and resolving within 1–3 days [16,18,19,20]. Some initial observations also indicate that those who receive a heterologous booster dose, regardless of the combination of vaccine types, can expect greater reactogenicity [14,17]. The exact reason behind this phenomenon requires further elucidation, but is likely associated with technological differences between particular vaccines and their varying mechanism of action. Nevertheless, further studies are needed to understand whether individuals factors, such as age, sex, BMI, and history of other vaccinations, may also influence reactogenicity to the booster dose.

This study aimed to assess the occurrence, frequency, and nuisance of adverse events following the administration of three doses of the mRNA COVID-19 vaccine and compare it to those recorded in individuals receiving two doses of the adenoviral vector vaccine further boosted with the mRNA vaccine. The use of pharmaceuticals to counteract the side effects of vaccinations was also recorded for the investigated groups, and their willingness to receive future COVID-19 vaccine doses if their administration would be recommended.

## 2. Materials and Methods

### 2.1. Data Collection

This research employed an anonymous, self-designed, and structured online questionnaire. The generalized invitations to fulfill it were emailed in 2021 to the academic and administrative staff of the Poznan University of Life Sciences, Cardinal Stefan Wyszyński University in Warsaw, and the Wroclaw Medical University, University Hospital in Wroclaw, and the Medical University of Gdańsk (Poland). The inclusion criteria for the study included age ≥18 years, receiving the two-doses homologous COVID-19 vaccination with either the mRNA (BNT16b2 by BioNTech/Pfizer, or mRNA-1273 by Moderna) or the adenoviral vector vaccine (AZD1222 by Oxford/AstraZeneca), receiving a third dose of the COVID-19 vaccine at least two weeks ago. During the study period, only mRNA vaccines were available in Poland as a booster dose (given as a full dose–30 µg–in the case of the BNT162b2 or a half dose–50 µg–in the case of the mRNA-1273). Therefore, two groups of individuals could be distinguished: vaccinated three times with the mRNA vaccine (3 × mRNA, homologously boosted); immunized with two doses of AZD1222 and boosted with the mRNA vaccine (2 × AZ + 1 × mRNA, heterologously boosted). The research aimed to specify:

- Types, number, and frequency of different adverse events (local and systemic) following the administration of each dose of COVID-19 vaccine;
- Duration of adverse events after administration of each dose of COVID-19 vaccine;
- Level of the nuisance of adverse events (if present) following administration of each dose of COVID-19 vaccine (measured with a 10-point Likert-type scale, where 1—low level of nuisance, 10—very high level of a nuisance);
- Demographical factors differentiating the frequency of adverse events and their nuisance level after the administration of the homologous and heterologous booster;
- Frequency and types of pharmaceuticals self-administered by participants to counteract the adverse events;
- Willingness to receive the potential future doses of the COVID-19 if recommended by the health authorities.

The following demographic data were collected from each participant: age; gender; height; weight (to calculate Body Mass Index); and history of COVID-19 prior to the third vaccine. Moreover, since the adult individuals who vaccinate against influenza may be more aware of the potential adverse events after vaccination, tolerate them better, and have a better understanding of repeated vaccination regimens [21,22], the history of influenza vaccine administration (received annually for at least two years; received the first influenza vaccine last year; never vaccinated) was recorded for each participant.

### 2.2. Statistical Analysis

PQStat (Poznań, Poland) was employed for data analysis. Because age and BMI did not meet the assumption of Gaussian distribution, while the nuisance level was measured by the ordinal Likert-type scale, a non-parametric Mann–Whitney U test was applied to compare the homologous (3 *×* mRNA) and the heterologous (2 *×* AZ + 1 *×* mRNA) group. The relationship between nuisance level and the number of side effects after first doses and a booster dose were assessed with Spearman’s correlation coefficient (Rs). In the case of nominal categorical variables, differences in frequencies were tested with Pearson’s χ^2^ test. A *p*-value < 0.05 was considered statistically significant.

## 3. Results

### 3.1. Demographic Characteristics

The demographic breakdown of both studied groups and the differences between them are summarized in Table 1. Both were represented chiefly by women. Individuals undergoing the 2 × AZ + 1 × mRNA vaccination scheme were significantly older, less frequently overweight and obese, and more often vaccinated against influenza at least once in their life (Table 1).

### 3.2. Occurrence of Vaccine Adverse Events

**Figure 1:**
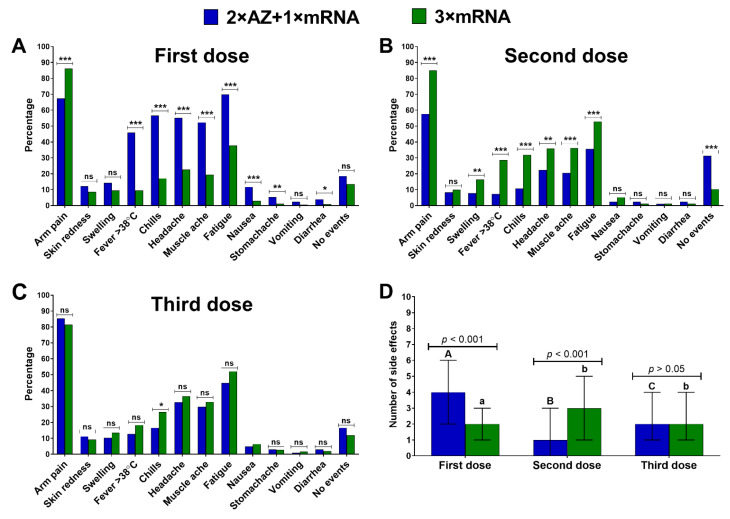
(**A**–**C**) Side effects reported in both groups after each vaccine dose. Asterisks indicate statistically significant differences in frequency between the groups (* *p* < 0.05; ** *p* < 0.01; *** *p* < 0.001; ns–not significant, *p* > 0.05); (**D**) The number of side effects (median and interquartile range) after each vaccine dose in the homologous and heterologous groups. Different capital letters indicate a significant difference between particular doses in the 2 × AZ + 1 × mRNA group, while different small letters indicate a significant difference between particular doses in the 3 × mRNA group (Kruskal–Wallis ANOVA with Dunn’s post-hoc test).

There were significant differences in the frequency and the number of side effects observed between both vaccination schemes (Figure 1). In the case of the first dose, side effects were more frequent and numerous after the AZD1222 administration than the mRNA vaccine, but the opposite was seen after the second dose. The frequency of side effects’ occurrence and their number after the third dose did not differ between the homologously and heterologously boosted groups, except for chills more often observed for individuals in the 3 × mRNA group. Arm pain and fatigue were the most common effects, regardless of the studied group and vaccine dose (Figure 1).

Various factors differentiated the frequency at which side effects occurred, as well as their number. In the homologously boosted group, the side effects associated with the third dose occurred more frequently in women than men (90.8 vs. 80.6%; Pearson’s χ^2^ test, *p* = 0.03). Moreover, women reported an increased number of side effects (median, IQR: 3, 1–5 vs. 2, 1–3; Mann–Whitney U test, *p* = 0.04). Although age did not affect the frequency of occurrence of side effects, their lower number was reported by individuals aged ≥50 years who received the homologous (median, IQR: 1, 1–2 vs. 2, 1–4; Mann–Whitney U test, *p* = 0.002) and heterologous booster (1, 1–3 vs. 2, 1–4, *p* = 0.02). In the homologously boosted group, higher BMI (≥25 kg/m^2^) was associated with a lower frequency of side effects occurrence (78.3 vs. 91.2%; Pearson’s χ^2^ test, *p* = 0.007), but not their number. Moreover, in the same group, individuals who received the influenza vaccine in the past for at least two years revealed the lowest frequency of side effects (69%), compared to those vaccinated for the first time in 2021 (91%), or never vaccinated against influenza (90%) (Pearson’s χ^2^ test, *p* = 0.007). No similar observations were made for the heterologously boosted group. A history of COVID-19 prior to the booster did not affect the frequency at which side effects occurred after the third dose in either vaccination group, but was related to their higher number in the case of the heterologously boosted individuals (median, IQR, 3, 1–5 vs. 2, 1–3; Mann–Whitney U test, *p* = 0.005). As presented in Table 2, the number of side effects after the third dose was positively correlated with the number reported after the first and second vaccine dose in both the homologous and heterologous group.

### 3.3. Level of Nuisance of Vaccine Adverse Events

**Figure 2:**
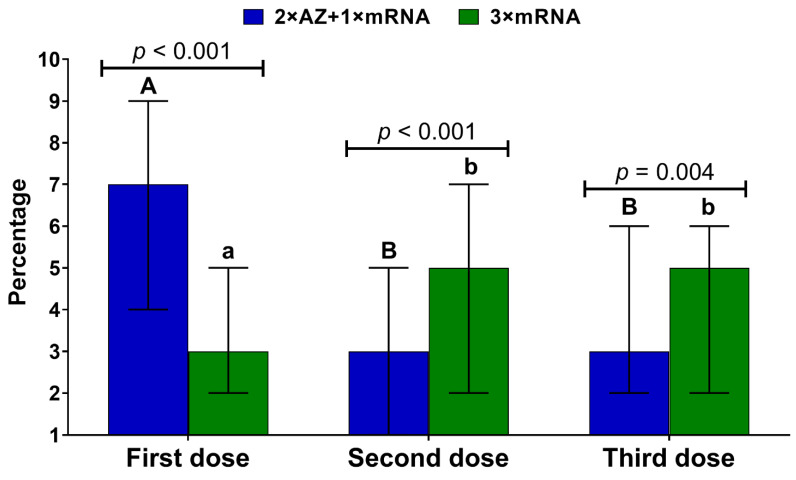
Side effects’ nuisance level (median and interquartile range) after each vaccine dose measured using a 10-point Likert scale in the homologous and heterologous groups. Different capital letters indicate a significant difference between particular doses in the 2 × AZ + 1 × mRNA group, while different small letters indicate a significant difference between specific doses in the 3 × mRNA group (Kruskal–Wallis ANOVA with Dunn’s post-hoc test).

Individuals from the 2 × AZ + 1 × mRNA and 3 × mRNA groups reported a different pattern of nuisance levels. For the former group, the highest level was observed after the first dose, with no difference between the second and third dose. In the case of the latter group, the highest nuisance level of side effects was reported after the second and third doses. The side effects after a booster dose were less burdensome in the heterologous group (Figure 2).

In the 3 × mRNA group, the nuisance level of adverse events after a booster dose was higher in women than men (median, IQR 5, 3–7 vs. 3, 1–6; Mann–Whitney U test, *p* = 0.003) and in individuals aged <50 compared to ≥50 years (5, 2–7 vs. 3, 1–5; Mann–Whitney U test, *p* = 0.03). In the heterologously boosted group, the nuisance level was higher for those who had COVID-19 prior to the third dose administration than in those without documented SARS-CoV-2 infection (median, IQR 4, 2–7 vs. 3, 1–6; Mann–Whitney U test, *p* = 0.02). In both booster groups, the nuisance level was not related to BMI and history of influenza vaccination. However, it correlated with the level of nuisance reported after previous doses, particularly the second one. Additionally, in the homologously boosted group, the number of side effects after the first and second vaccine dose was associated with the increased nuisance level after a third dose (Table 3).

### 3.4. Pharmaceuticals Used to Counteract Vaccine Adverse Events

The frequency of pharmaceutical use to counteract the vaccination side effects in the 2 × AZ + 1 × mRNA was higher after the first dose than in the 3 × mRNA group (62.4 vs. 26.6%; Pearson’s χ^2^ test, *p* < 0.001), lower after the second dose (27.8 vs. 42.9%; Pearson’s χ^2^ test, *p* < 0.001) and similar after the third dose (36.1 vs. 40.1%; Pearson’s χ^2^ test, *p* > 0.05). Among those experiencing side effects, a considerable number declared not having used any medications (23–47% in the 2 × AZ + 1 × mRNA group and 38–53% in the 3 × mRNA group). Other individuals mostly selected paracetamol. A considerably lower percentage used ibuprofen, followed by metamizole and acetylsalicylic acid (Table 4).

### 3.5. Willingness to Receive Future COVID-19 Vaccine Doses

The vast majority (93.0%) of all individuals taking part in this survey declared a willingness to receive further vaccine doses if recommended in the future, 4.8% were unsure about it, while only 2.2% (*n* = 10) declared they were unwilling. The frequency of unwillingness to receive another dose did not differ between the two groups of vaccination schemes and was not differentiated by the demographical variables considered in this study. However, the individuals unwilling to receive it reported a greater number of side effects following the third vaccine dose (median, IQR 6, 4–9 vs. 2, 1–4; Mann–Whitney U test, *p* = 0.004) and their higher nuisance level (median, IQR: 10, 8–10 vs. 4, 2–6; Mann–Whitney U test, *p* = 0.002).

## 4. Discussion

This study provides an overview of the side effects of COVID-19 vaccination, their frequency, number, and nuisance in individuals receiving the homologous and the heterologous booster dose. The reported observations may be especially important for those regions which, due to various reasons, are less vaccinated and will pursue booster strategies in the future [1,23].

The higher number of adverse events recorded after the first dose of the AZD1222 vaccine than the mRNA vaccine and the opposite seen after a second dose closely reflect the results of the pre-authorization clinical trials. It is likely related to technological differences between the mRNA and adenoviral vector vaccines [24,25,26,27,28]. Although the 2 × AZ + 1 × mRNA and 3 × mRNA groups differed significantly in the frequency and number of side effects after the first and second vaccine dose, no relevant differences were seen following the booster administration. Moreover, the frequency of particular adverse events after a third vaccine dose was similar in both groups. It contradicts the previous findings indicating that heterologous vaccination, in the case of second and a third dose, is related to greater reactogenicity [14,29]. Interestingly, the present study reports a higher level of the self-assessed nuisance of side effects in the homologously boosted group. Moreover, this group declared a slightly (by 4%) higher use of pharmaceuticals to counteract the side effects after administration of the booster dose. Altogether this indicates that, at least in the groups demographically similar to those included in our research, a heterologous boosting strategy may offer some advantage in terms of how vaccinated individuals cope with side effects.

Regardless of the vaccination group and vaccine dose, individuals choosing pharmaceuticals to counteract the side effects predominantly used paracetamol. As evidenced during the Phase 1/2 clinical trial of the AZD1222 vaccine, the prophylactic use of paracetamol did not affect immunogenicity [26]. Experience with other immunizations, e.g., hepatitis B vaccination, demonstrated that the therapeutic use of paracetamol is safe and does not affect immune response [30]. Therefore, some health authorities have recommended its use to relieve the side effects following COVID-19 vaccination, such as fever, chills, or headache [31]. Ibuprofen, the second most often used pharmaceutical by individuals considered in the present study, has a specific anti-inflammatory mechanism of action through inhibition of the cyclooxygenases 1 and 2. Some in vitro studies have shown that it can blunt IgM and IgG production in activated human B cells [32]. On the other hand, it is unlikely that the occasional use of ibuprofen may significantly affect the response to immunization. In addition, one study showed that, compared to individuals using paracetamol, the average of side effects following mRNA vaccination, such as fever and headache, was significantly shorter in those who used non-steroidal anti-inflammatory drugs, including ibuprofen [33].

The present study indicates that those who experience a broader spectrum of side effects and greater nuisance levels following the primary COVID-19 vaccination are likely to expect more adverse events after both a homologous and the heterologous booster dose. This suggests that, although reactogenicity may differ between vaccine doses or particular vaccine types, some individuals may be more vulnerable to the experience of vaccination. One should note that this may not always be necessarily related to the vaccine constituents. Analysis of adverse events recorded in COVID-19 vaccine trials indicated that the rates of nocebo responses, i.e., unwanted effects seemingly elicited by placebo administration, are substantial [34]. This may be due to anxiety associated with intervention or misconceptions about potential side effects [35]. Therefore, informing patients on vaccine adverse events and the nocebo phenomenon may help reduce the actual number of these events following vaccination and decrease vaccine hesitancy [34,36,37,38]. In addition, the present study suggests that those who experienced a higher frequency and severity of adverse events after the initial COVID-19 vaccination protocol should be informed of the potentially increased risk of side effects of a booster dose. It also shows that women, individuals without overweight or obesity, and those who do not receive regular influenza vaccine may experience side effects more frequently or more numerously after the homologous mRNA booster vaccine. Moreover, both the homologous and heterologous booster vaccination may increase the number of side effects in those aged <50 years. These observations may be valuable for accurate and honest communication with patients, which is pivotal in improving their overall experience with vaccination and trust in representatives of public health [39,40,41,42].

Interestingly, the present study also found that individuals who receive a regular influenza vaccination experience a lower frequency of side effects following an mRNA booster vaccine dose. Throughout the COVID-19 pandemic, different studies have indicated that the influenza vaccination may decrease the severity and mortality of SARS-CoV-2 infection [43,44]. This suggests that the influenza vaccination may stimulate the epigenetic and metabolic reprogramming of the innate immune response in the process of so-called trained immunity, leading to a more robust and targeted response to a nonspecific stimulus, such as other pathogens [45,46,47]. It should be stressed that our study cannot discern a mechanism behind the lower reactogenicity of the homologous mRNA booster vaccine in individuals who were regularly receiving the influenza vaccination. However, it advocates for further observational studies to understand whether adults with a broader vaccination history are experiencing adverse events less frequently

Considering that SARS-CoV-2 is unlikely to be eradicated and will undergo further evolution, while humoral responses are waning several months after COVID-19 vaccination, recommendation of additional booster doses may be necessary for the future. At present, there are not enough data to understand whether they should be given annually or at a lesser frequency [48]. Importantly, the present study shows that most of those who received a booster dose are also willing to be vaccinated with potential future doses. Those who declared no interest reported a high number of adverse events and their increased nuisance level after receiving the third dose. This highlights that past experiences play a role in vaccine perception and shape future decisions regarding vaccination, as already documented in previous research [22,49].

Study limitations must be stressed. Firstly, since the design of this study was based on anonymous self-reporting, the volunteer bias cannot be excluded. Secondly, the considered groups revealed differences in selected demographical characteristics. This was due to different recommendations on the administration of vector and mRNA vaccines for various occupational and age groups. Although the reported data are relevant to predict the frequency, number, and nuisance of side effects in individuals scheduled to receive homologous and heterologous booster vaccine doses who share similar characteristics to those included in the present study (e.g., younger in the case of the former group, and older in the case of the latter), the extrapolation to other populations should be made cautiously. Thirdly, the side effects were reported retrospectively and anonymously two weeks after receiving the booster vaccine dose by the surveyed individuals, excluding the possibility of verifying the data on objective grounds. Fourth, the present study compared only groups receiving the mRNA vaccine as a homologous or heterologous booster, and it is unknown whether these results can translate to different combinations of vaccine types. Moreover, the willingness to receive potential future COVID-19 vaccine doses may not always be reflected in actual decisions, as they can be affected by various factors, e.g., epidemiological situation and personal experiences with COVID-19. According to the study conducted in September 2021, immediately before the booster dose was recommended in Poland, 71% of Poles who completed an initial vaccination protocol were willing to receive it [48]. Therefore, it was expected that at least 13 million people would be vaccinated with a booster dose, although by the end of March 2022, their number reached only 11.75 million [50].

## 5. Conclusions

The present study indicates that individuals undergoing an initial COVID-19 vaccination scheme with two doses of the adenoviral vector vaccine and the mRNA vaccine reveal different frequency patterns and number of side effects, but they may not vary after the administration of homologous and heterologous booster mRNA vaccinations. Nevertheless, individuals receiving homologous booster doses may experience a higher nuisance of side effects and need to use pharmaceuticals, such as paracetamol. Women, individuals with BMI < 25 kg/m^2^, and aged <50 years who follow this vaccination scheme may be more prone to the side effects of the third dose. The findings also highlight the potential protective effect of the influenza vaccination in this regard. Individuals who experience a higher number and nuisance of side effects after the second dose of adenoviral or mRNA vaccine are more likely to have similar experiences following the administration of the homologous or heterologous booster dose of the mRNA vaccine. The reported observations may be valuable in shaping accurate and honest communication with vaccinated individuals, especially in regions that have yet to pursue booster strategies.

## Figures and Tables

**Table 1:** The demographic characteristics of the studied groups of vaccinated individuals.

Parameter	All (*n* = 457)	2 × AZ + 1 × mRNA (*n* = 205)	3 × mRNA (*n* = 252)	*p*-Value
**Gender**	65.6 (300)/	66.3 (136)/	65.1 (164)/	>0.05
Women/men, % (*n*)	34.4 (157)	33.7 (69)	34.9 (88)	
**Age** (years), mean ± SD	35.8 ± 14.9	44.6 ± 12.4	28.7 ± 12.8	<0.001
(min–max)	(19–75)	(20–69)	(19–75)	
≥50 years, % (*n*)	22.5 (103)	34.1 (70)	13.1 (33)	<0.001
**BMI** (kg/m^2^), mean ± SD	24 ± 4.5	23 ± 4.3	24.8 ± 4.5	<0.001
(min–max)	(16.5–46.3)	(16.5–44.2)	(17.5–46.3)	
Underweight, % (*n*)	6.6 (30)	9.3 (19)	4.4 (11)	0.036
Normal weight, % (*n*)	58.4 (267)	65.4 (134)	52.8 (133)	0.007
Overweight, % (*n*)	25.4 (116)	19 (39)	30.5 (77)	0.005
Obese, % (*n*)	9.6 (44)	6.3 (13)	12.3 (31)	0.032
**Influenza vaccination status**				
Unvaccinated, % (*n*)	57.5 (263)	67.3 (138)	49.6 (125)	<0.001
Vaccinated for the first time in 2021	17.5 (80)	10.3 (21)	23.4 (59)	<0.001
Vaccinated annually at least twice	25 (114)	22.4 (46)	27 (68)	<0.001

**Table 2:** The Spearman’s correlation coefficient calculated between a number of side effects observed after particular vaccine doses in the 2 × AZ + 1 × mRNA and 3 × mRNA groups.

Parameter	Number of Side Effects after 3rd Dose	
	2 × AZ + 1 × mRNA	3 × mRNA
Number of side effects after 1st dose	0.37 (***)	0.37 (***)
Number of side effects after 2nd dose	0.41 (***)	0.55 (***)

*** *p* < 0.001.

**Table 3:** The Spearman’s correlation coefficient calculated between a number of side effects and nuisance level primary vaccine doses and nuisance level of side effects after the third dose in the homogenously and heterologously boosted groups.

Parameter	Vaccine Dose	Level of Nuisance after 3rd Dose	
		2 × AZ + 1 × mRNA	3 × mRNA
Number of side effects	1st dose	0.37 (ns)	0.18 (**)
	2nd dose	0.07 (ns)	0.41 (***)
Level of nuisance of side effects	1st dose	0.22 (**)	0.13 (ns)
	2nd dose	0.32 (***)	0.52 (***)

Ns–not significant, *p* > 0.05; ** *p* < 0.01; *** *p* < 0.001.

**Table 4:** The frequency of pharmaceutical use in individuals experiencing side effects after vaccination.

Pharmaceutical	Vaccine Scheme	First Dose	*p*-Value	Second Dose	*p*-Value	Third Dose	*p*-Value
None, despite side effects	2 × AZ + 1 × mRNA	23.2	0.003	47.0	>0.05	41.2	>0.05
	3 × mRNA	53.2		38.0		40.7	
Paracetamol	2 × AZ + 1 × mRNA	57.3	<0.001	37.0	<0.001	40.5	>0.05
	3 × mRNA	32.9		42.7		38.1	
Ibuprofen	2 × AZ + 1 × mRNA	11.9	>0.05	10.0		13.7	>0.05
	3 × mRNA	10.8		16.2		18.0	
Metamizole	2 × AZ + 1 × mRNA	4.9	>0.05	3.0	>0.05	2.3	>0.05
	3 × mRNA	2.5		3.1		1.5	
Acetylsalicylic acid	2 × AZ + 1 × mRNA	2.7	>0.05	3.0	>0.05	2.3	>0.05
	3 × mRNA	0.6		0.0		1.5	

## Data Availability

The data presented in this study are available from the corresponding author on reasonable request.
